# Relationship between self-reported sleep quality and metabolic syndrome in general population

**DOI:** 10.1186/1471-2458-14-562

**Published:** 2014-06-05

**Authors:** Noriyuki Okubo, Masashi Matsuzaka, Ippei Takahashi, Kaori Sawada, Satoshi Sato, Naoki Akimoto, Takashi Umeda, Shigeyuki Nakaji

**Affiliations:** 1 Department of Social Medicine, Hirosaki University Graduate School of Medicine, 5, Zaifu-cho, Hirosaki, Aomori 036-8562, Japan; 2 Department of Medical Informatics, Hirosaki University Graduate School of medicine, 5, Zaifu-cho, Hirosaki, Aomori 036-8562, Japan; 3 Department of Faculty of Pharmacy, Meijo University, Yagotoyama 150, Tenpaku-ku, Nagoya, Aichi 468-8503, Japan

**Keywords:** Metabolic syndrome, Obesity, Pittsburgh sleep quality index, Sleep quality, General population

## Abstract

**Background:**

To examine an association between self-reported sleep quality determined by Pittsburgh sleep quality index (PSQI) and metabolic syndrome.

**Methods:**

This study was designed as cross-sectional study. Participants were 1481 adults aged 20 years and above from general population (549 males and 932 females). We assessed the global sleep quality by PSQI. PSQI consists of 7 elements, i.e. subjective sleep quality, sleep latency (prolonged sleep onset time), sleep duration, habitual sleep efficiency (proportion of hours slept to hours spent in bed), sleep disturbance (interruption of sleep), use of sleep medication and daytime dysfunction (trouble staying awake while engaging in social activity). Any participants with score of 6 or more are diagnosed to have sleep disorder. We also assessed the above 7 elements, which consisted of a four-grade system (i.e. 0, 1, 2, 3). Metabolic syndrome consisted of abdominal obesity, hypertension, impaired glucose tolerance and dyslipidemia. Diagnosis of metabolic syndrome was done when the participants have abdominal obesity and meet two or more other components. All analyses were adjusted by age, drinking habit, smoking habit, working hours, exercise habit and depression.

**Results:**

Fifty-two male participants (9.5%) and 133 female (14.3%) scored 6 or more points in global PSQI score. The global PSQI score, sleep latency score and sleep disturbance score of participants with metabolic syndrome were higher level than those without the condition (p < 0.001, p = 0.009, p = 0.025 for male and p < 0.001, p < 0.001, p = 0.002 for females, respectively). The odds ratio of metabolic syndrome among participants with PSQI score of 6 or more points were 2.37 (95% confidence interval: 1.23-4.58) for males and 2.71 (1.45-5.07) for females in contrast to those with 5 or less points. The odds ratio of metabolic syndrome with sleep latency score of 2 was 2.65 (1.14-6.15) for male and 3.82 (1.81-8.09) for females in contrast with those of 0. The odds ratio of metabolic syndrome with sleep disturbance score of 1 was 1.76 (1.09-2.86) for males and 2.43 (1.26-4.69) for females in contrast with those of 0.

**Conclusions:**

Global PSQI score and its components (especially, sleep latency and sleep disturbance) were associated with metabolic syndrome.

## Background

Metabolic syndrome, which consists of a complex combination of abdominal obesity, hypertension, impaired glucose tolerance and dyslipidemia is a large health problem around the world. This medical disorder increases morbidity and mortality of cardiovascular diseases and stroke, for it raises risks of atherosclerosis [1-3]. To decrease the mortality or morbidity of cardiovascular diseases and stroke, it is important to prevent and to improve their risk factors including metabolic syndrome. It has been thought that metabolic syndrome is caused by a nutrient imbalance with impaired eating habits and low consumption of calories due to lack of exercise. Thus, various public organizations and medical institutions have been providing advice to encourage appropriate dietary intake and physical exercise to the public.

Associations of sleep duration with obesity, diabetes mellitus, hypertension, atherosclerosis and cardiovascular diseases have been reported [4-8]. A role of sleep is not only rest of body but also suppression of blood pressure and glucose tolerance by decreasing the secretion of catecholamine and cortisol [9], which then leads to prevention of metabolic syndrome and other obesity-related diseases.

Hall et al. have reported that those who have slept 7 to 8 hours per night have had the lowest morbidity of metabolic syndrome, and those who have slept longer or shorter have shown an increase risk of metabolic syndrome (the so-called U-shaped associations) [10]. Moreover, short and long sleep duration has been associated with higher mortality of cardiovascular diseases [11,12]. However, assessment of sleep only by its duration is not enough. Some previous studies have suggested that status of sleep has quantitative and qualitative aspects [13,14]. Actually, some studies have reported that sleep quality is associated with overweight and metabolic syndrome [15,16], but the limited number of the studies cannot provide good evidence of it. Also, insufficient adjustment of confounding factors such as gender and lifestyles including drinking and exercise habit also give us controversy.

For better evidence, we analyzed data separately by gender and adjusted by age, and other plausible confounding factors to examine the relationship of sleep and its components with metabolic syndrome among Japanese general population.

## Methods

### Participants

Participants were 1552 individuals from the general population who participated in the Iwaki Health Promotion Project from 2007 to 2010 in Aomori Prefecture, located in northern Japan. The project was a screening project for the residents, and this study was designed as a population-based cross-sectional research. Any participants with missing data, steroid intake, clinical history of malignant diseases or psychotic illness were excluded from the study. Thus, the number of participants who were eligible for statistical analysis included 1481 participants (549 males and 932 females). Ages of the participants were between 20 and 80 years old. The contents and purpose of this study were thoroughly explained to the participants prior to the study, and written consent was obtained from all of the participants.

Approval for the study was obtained from the Ethics Committee of Hirosaki University Graduate School of Medicine.

#### Measures and definitions

Self-reported questionnaires were sent to the participants prior to the investigation and were collected on the day of investigation. In the questionnaires, participants were asked about their gender, age, smoking habit, drinking habit, exercise habit, working hours per week, PSQI, presence of depression, medical history of malignant diseases or psychotic illness and any medications which they are currently taking (hypnotic drugs, steroid, antidyslipidemic drugs, antihypertensive drugs, antidiabetic drugs). We used the Center for Epidemiologic Studies Depression (CES-D) scale for an index of depression. CES-D scale was established in 1977 by Radloff to evaluate presence and grades of depression. It consists of twenty items with sixty points as a perfect score, and those who scored more than sixteen points are diagnosed to be depressive [17]. Exercise habit was determined whether or not they exercised regularly. Working hours per week included that of main job and of added job.

We assessed sleep quality of the participants by the Pittsburgh Sleep Quality Index (PSQI) scale [18]. It is a widely used measure of sleep quality and is well validated, reliable and readily completed by most individuals. PSQI consists of 7 elements: subjective sleep quality (a subjective feeling of satisfaction at daily sleep), sleep latency (prolonged sleep onset time), sleep duration, habitual sleep efficiency (proportion of hours slept to total hours in bed), sleep disturbance (interruption of sleep), use of sleep medication, and daytime dysfunction (trouble staying awake while engaging in social activity) with total score of 21. Any participants with score of 6 or more are diagnosed to have sleep disorder [18]. We assessed not only global PSQI score but also the above 7 components, which consisted of the four-grade system (i.e. 0, 1, 2, 3) [18]. Subjective sleep quality was assessed 0–3 points (0 - very good sleep quality; 1 - fairly good sleep quality; 2 - fairly bad sleep quality; 3 - very bad sleep quality). Sleep latency was assessed 0–3 points (minutes required to go to sleep each night and frequency of getting to sleep within 30 minutes). Sleep duration was assessed 0–3 points (0 - sleep duration >7 hours; 1 - 6- ≤ 7 hours; 2 - 5- < 6 hours; 3 - <5 hours). Habitual sleep efficiency was assessed 0-3points (proportion of hours slept to hours spent in bed, 0 - ≥85%; 1 - 75-84%; 2 - 65-74%; 3 - <65%). Sleep disturbance was assessed 0-3points (having trouble sleeping because of some reasons and the frequency of those). Use of sleep medication was assessed 0-3points (0 - not use during the past month; 1 - less than once a week; 2 - once or twice a week; 3 - three or more times a week). Daytime dysfunction was assessed 0–3 points (frequency of having trouble staying awake while driving, eating meals, or engaging in social activity and frequency of having trouble keeping up enough enthusiasm to get things done).

We measured height, weight, waist circumference and blood pressure as physical data of the participants. Body mass index (BMI) was calculated as weight (kg) divided by height in meters squared (m^2^). The waist circumference was measured with participants standing up straight, at a navel level with light expiration. When adiposity was remarkable, we measured waist circumference at the middle point of costal inferior border and anterior intestinal portal osteophyte. Blood pressure was measured as systolic and diastolic blood pressure on the arm in a seated position by an automatic sphygmomanometer.

We also collected venous blood samples from the participants when they were fasting on the day of investigation. Determination of serum concentration of triglyceride, high density lipoprotein cholesterol (HDL-C) and glucose were performed with enzyme assay by the Mitsubishi Chemical Medience Corporation.

Metabolic syndrome was defined by eight medical societies (Committee to Evaluate Diagnostic Standards for Metabolic Syndrome) in Japan [19]. Metabolic syndrome consists of four criteria as follows: (1) abdominal obesity, waist circumference of 85 cm or greater in male, 90 cm or greater in female; (2) hypertension, systolic blood pressure of 130 mmHg or greater, or diastolic blood pressure of 85 mmHg or greater, or use of antihypertensive drugs; (3) impaired glucose tolerance, serum glucose of 110 mg/dL or greater, or use of antidiabetic drugs; and (4) dyslipidemia, serum triglyceride of 150 mg/dL or greater, or serum HDL-C of less than 40 mg/dL, or use of antidyslipidemic drugs. Diagnosis of metabolic syndrome was done when the participants have abdominal obesity and meet two or more other criteria.

### Statistical analysis

All statistical analyses were conducted separately by gender because some previous studies had suggested gender differences of sleep outcomes [13]. Unpaired t-test was used to determine differences of age, BMI, sleep duration and global PSQI score. Chi-square test was used to test differences of drinking habit, smoking habit, exercise habit, metabolic syndrome, abdominal obesity, hypertension, impaired glucose tolerance and dyslipidemia. Analysis of covariance (ANCOVA) for continuous variables was performed to determine any significant differences among groups of non-metabolic syndrome and metabolic syndrome after adjusted by age, drinking habit, smoking habit, working hours per week, exercise habit and depression. Logistic regression analysis was conducted to assess relationships of sleep disorder diagnosed with global PSQI score and other 7 elements with metabolic syndrome after adjusted by plausible confounding factors such as age, lifestyles including smoking habit, drinking habit, exercise habit, working hours per week and depression. Odds ratios were calculated from logistic regression analysis with 95% confidence intervals.

All analyses were performed using SPSS 12.0 J for Windows, and p < 0.05 was considered to be statistically significant.

## Results

Characteristics of the participants are shown in Table 1. Of 1481 participants, 549 (37.1%) were males and 932 (62.9%) were females. Mean of BMI in males was 23.6 kg/m^2^ and 22.9 kg/m^2^ in females, and was thus significantly higher in males than females. Mean of sleep duration was 461 minutes in males and 430 minutes in females, and thus significantly longer in males than females. Proportion of the participants with drinking habit and smoking habit were greater in males than in females. Prevalence of depression was lower in males than in females. Prevalence of metabolic syndrome, abdominal obesity, hypertension, impaired glucose tolerance and dyslipidemia was greater in males than in females. Global PSQI score was significantly higher in females than males. Among all participants, 52 of males (9.5% of total males) and 133 of females (14.3% of total females) scored 6 or more.

**Table 1:** Characteristics of participants

	**Male (n = 549)**	**Female (n = 932)**	**p value**
Age, y	57.1 ± 14.4	57.9 ± 13.6	0.246
BMI, kg/m^2^	23.6 ± 2.9	22.9 ± 3.4	<0.001
Sleep duration, min/day	461 ± 76	430 ± 68	<0.001
Current drinker	402 (73.2)	222 (23.8)	<0.001
Current smoker	198 (36.1)	89 (9.5)	<0.001
Exercise habits	156 (28.4)	244 (26.2)	0.349
Depression	83 (15.1)	218 (23.4)	<0.001
Metabolic syndrome	105 (19.1)	63 (6.8)	<0.001
Abdominal obesity	262 (47.7)	187 (20.1)	<0.001
Hypertension	322 (58.7)	476 (51.1)	0.005
Impaired fasting glucose	79 (14.4)	70 (7.5)	<0.001
Dyslipidemia	154 (28.1)	190 (23.4)	0.001
Global PSQI score, points	2.8 ± 2.0	3.4 ± 2.3	<0.001
0-1	152 (27.7)	191 (20.5)	
2-3	231 (42.1)	364 (39.1)	
4-5	114 (20.8)	244 (26.2)	
6-7	34 (6.2)	80 (8.6)	
8-9	12 (2.2)	31 (3.3)	
10-11	4 (0.7)	14 (1.5)	
12-13	2 (0.4)	5 (0.5)	
14-15	0 (0.0)	3 (0.3)	
16-21	0 (0.0)	0 (0.0)	

Data are presented as mean ± standard deviation or number (%).

The chi-square statistical test for nominal variables and one-way analysis of variance for continuous variables were performed to assess whether there were significant differences among the groups stratified by sex.

*Abbreviations*: BMI, body mass index.

Table 2 shows the significant differences among the groups of non-metabolic syndrome and metabolic syndrome. In males with metabolic syndrome, the means of global PSQI score, sleep latency score, sleep duration score and sleep disturbance score were significantly higher than those without metabolic syndrome. In females with metabolic syndrome, the means of global PSQI score, sleep latency score, habitual sleep efficiency score, sleep disturbance score and use of sleep medication score were significantly higher compared to those without metabolic syndrome.

**Table 2:** Comparison of each PSQI score between the groups of without metabolic syndrome and with metabolic syndrome

	**Without MetS**	**With MetS**	**p value**
**Male**			
Global PSQI score	2.69 ± 0.09	3.44 ± 0.19	<0.001
Subjective sleep quality	0.76 ± 0.03	0.87 ± 0.06	0.066
Sleep latency	0.43 ± 0.03	0.63 ± 0.07	0.009
Sleep duration	0.49 ± 0.03	0.69 ± 0.07	0.009
Habitual sleep efficiency	0.02 ± 0.01	0.05 ± 0.02	0.061
Sleep disturbance	0.54 ± 0.02	0.67 ± 0.05	0.025
Use of sleep medication	0.10 ± 0.02	0.11 ± 0.05	0.744
Daytime dysfunction	0.36 ± 0.03	0.41 ± 0.06	0.408
**Female**			
Global PSQI score	3.27 ± 0.07	4.78 ± 0.28	<0.001
Subjective sleep quality	0.81 ± 0.02	0.90 ± 0.08	0.081
Sleep latency	0.53 ± 0.03	1.04 ± 0.10	<0.001
Sleep duration	0.78 ± 0.03	0.88 ± 0.10	0.323
Habitual sleep efficiency	0.01 ± 0.01	0.21 ± 0.03	<0.001
Sleep disturbance	0.57 ± 0.02	0.79 ± 0.07	0.002
Use of sleep medication	0.16 ± 0.02	0.45 ± 0.08	0.001
Daytime dysfunction	0.41 ± 0.02	0.51 ± 0.08	0.231

Data are presented as mean ± standard error.

One-way analysis of covariance for continuous variables were performed to assess whether there were significant differences among the groups of non-metabolic syndrome and metabolic syndrome after adjusted for age, current drinking status, current smoking status, working hours per a week, exercise habits and depression.

*Abbreviations*: Mets, metabolic syndrome. PSQI, Pittsburgh sleep quality index.

The results of logistic regression analysis on associations between sleep disorder (global PSQI and its components) and metabolic syndrome after adjusted for age, lifestyle and depression are shown in Tables 3 and 4. In males, odds ratio of metabolic syndrome among those with the global PSQI score of 6 or more was 2.37 in contrast with those whose score was 5 or less. In females, odds ratio of metabolic syndrome among those with the global PSQI score of 6 or more was 2.71 in contrast with those with global PSQI score of 5 or less. Our results showed no associations between subjective sleep quality and metabolic syndrome in males and females. In males, the odds ratio of it among those with sleep latency score of 2 was 2.65 in contrast with those with 0. In females, the odds ratios of it among those with sleep latency score of 2 and 3 were 3.82 and 5.95, respectively, in contrast with those of 0. In males, the odds ratios among those with sleep duration score of 1 (6–7 hours/day) and 3 (<5 hours/day) were 1.89 and 14.08, respectively, in contrast with those of 0 (>7 hours/day). Results in females showed no association between sleep duration and metabolic syndrome. Males and females had no association between habitual sleep efficiency and metabolic syndrome. In males, the odds ratio among those with sleep disturbance score of 1 was 1.76 in contrast with those of 0. In females, the odds ratios among those with sleep disturbance score of 1 and 2 were 2.43 and 3.84, respectively, in contrast with those of 0. Males had no association between use of sleep medication score and metabolic syndrome. In females, the odds ratios among those with use of sleep medication score of 1 (1 time/week) and 2 (≥3 times/week) were 3.81 and 3.10, respectively, in contrast with those of 0 (no use of medication). Males had no association between daytime dysfunction and metabolic syndrome. In females, the odds ratio among those with daytime dysfunction score of 3 was 6.27 in contrast with those of 0.

**Table 3:** Odds ratio of metabolic syndrome by PSQI and its components in males

	**Score**	**n**	**OR**	**95% CI**	**p value**
Global PSQI score	≤5	497	1.00	(reference)	
	≥6	52	2.37	(1.23-4.58)	0.010
Subjective sleep quality	0	173	1.00	(reference)	
	1	323	1.14	(0.69-1.88)	0.603
	2	52	1.88	(0.87-4.07)	0.107
	3	1	-	-	
Sleep latency	0	353	1.00	(reference)	
	1	152	1.54	(0.95-2.51)	0.082
	2	29	2.65	(1.14-6.15)	0.023
	3	15	1.89	(0.58-6.21)	0.293
Sleep duration	0	329	1.00	(reference)	
	1	151	1.89	(1.14-3.13)	0.014
	2	66	1.65	(0.81-3.38)	0.172
	3	3	14.08	(1.21-163.85)	0.035
Habitual sleep efficiency	0	537	1.00	(reference)	
	1	11	1.90	(0.52-6.97)	0.334
	2	1	-		
	3	0	-		
Sleep disturbance	0	251	1.00	(reference)	
	1	285	1.76	(1.09-2.86)	0.022
	2	12	2.44	(0.64-9.22)	0.190
	3	1	-		
Use of sleep medication	0	528	1.00	(reference)	
	1	3	0.00	(0.00- )	0.999
	2	3	1.40	(0.12-16.48)	0.791
	3	15	1.17	(0.36-3.75)	0.797
Daytime dysfunction	0	375	1.00	(reference)	
	1	152	1.61	(0.99-2.62)	0.055
	2	17	0.30	(0.18-2.37)	0.255
	3	5	1.75	(0.18-16.84)	0.628

Data are presented as adjusted odds ratio (95% Confidence Interval) of metabolic syndrome.

Logistic regression analysis was conducted after adjusted for age, current drinking status, current smoking status, working hours per a week, exercise habits and depression.

*Abbreviations*: OR, odds ratio. CI, confidence interval. PSQI, Pittsburgh sleep quality index.

**Table 4:** Odds ratio of metabolic syndrome by PSQI and its components in females

	**Score**	**n**	**OR**	**95% CI**	**p value**
Global PSQI score	≤5	799	1.00	(reference)	
	≥6	133	2.71	(1.45-5.07)	0.002
Subjective sleep quality	0	290	1.00	(reference)	
	1	530	1.41	(0.77-2.58)	0.263
	2	108	1.55	(0.59-4.08)	0.378
	3	4	2.96	(0.26-34.11)	0.385
Sleep latency	0	555	1.00	(reference)	
	1	261	1.65	(0.86-3.16)	0.131
	2	85	3.82	(1.81-8.09)	<0.001
	3	31	5.95	(2.17-16.34)	0.001
Sleep duration	0	411	1.00	(reference)	
	1	324	0.96	(0.50-1.85)	0.904
	2	186	1.57	(0.81-3.06)	0.185
	3	11	1.20	(0.13-10.91)	0.870
Habitual sleep efficiency	0	915	1.00	(reference)	
	1	11	3.96	(0.96-16.34)	0.057
	2	4	-		0.999
	3	2	10.42	(0.61-176.68)	0.105
Sleep disturbance	0	412	1.00	(reference)	
	1	492	2.43	(1.26-4.69)	0.008
	2	27	3.84	(1.17-12.65)	0.027
	3	1	-		
Use of sleep medication	0	861	1.00	(reference)	
	1	18	3.81	(1.15-12.68)	0.029
	2	10	1.12	(0.13-9.86)	0.916
	3	43	3.10	(1.32-7.30)	0.009
Daytime dysfunction	0	609	1.00	(reference)	
	1	263	1.12	(0.61-2.06)	0.710
	2	52	0.75	(0.16-3.52)	0.718
	3	8	6.27	(1.24-31.79)	0.027

Data are presented as adjusted odds ratio (95% Confidence Interval) of metabolic syndrome.

Logistic regression analysis was conducted after adjusted for age, current drinking status, current smoking status, working hours per a week, exercise habits and depression.

*Abbreviations*: OR, odds ratio. CI, confidence interval. PSQI, Pittsburgh sleep quality index

## Discussion

In previous studies, assessment of sleep duration has been mainly used to evaluate the status of sleep. However, measuring sleep duration only is insufficient for understanding global sleep status, which consist of not only sleep duration but also quality and other various factors. These sleep-related factors include quantitative and qualitative variables, which has made sleep status difficult to understand. Taking both aspects into consideration, our study used PSQI scale to evaluate global sleep status. We examined the associations of metabolic syndrome with global PSQI scale and its components (sleep quality, sleep latency, sleep duration, habitual sleep efficiency, sleep disturbance, use of sleep medication and daytime dysfunction). Our results showed significant associations of metabolic syndrome with global PSQI score, poor sleep quality, prolonged sleep latency, short sleep duration, sleep disturbance and daytime dysfunction. So far no studies have reported the association between metabolic syndrome and PSQI components. Especially, global sleep status that included sleep latency, habitual sleep efficiency, sleep disturbance and daytime dysfunction has rarely received attention in scientific research. Thus, the current study could provide novel findings which suggest the relationship between sleep status and metabolic syndrome.

Kazman et al. have reported that there has been no association between global PSQI score and metabolic syndrome [20]. On the other hand, some studies have reported that the global PSQI score has been associated with metabolic syndrome [15,16]. Thus, the association between sleep and metabolic syndrome has various aspects. One of the possible reasons for such variations is that participants of those studies include clinical patients and healthy individuals with limited range of age. Additionally, in previous studies, the confounding factors such as lifestyles including drinking habits and exercise habit have not been sufficiently adjusted. Data obtained in this study was analyzed separately by gender and adjusted by age, drinking habit, smoking habit, exercise habit and depression. The participants of this study were all adults from Japanese general population, and the mean of global PSQI score with metabolic syndrome and non-metabolic syndrome was 5 or less (no sleep disorder). However, we also indicated that global PSQI score with metabolic syndrome was higher than those with non-metabolic syndrome. Consequently, those who scored high global PSQI score would be at high risk of metabolic syndrome even though they have no sleep disorder.

What we could underline was that we examined the correlation between PSQI components and metabolic syndrome. Many previous studies assessed sleep status by global PSQI score only, whereas our study assessed it by PSQI components as well as global score. This study indicated the PSQI components including poor sleep quality, prolonged sleep latency, short sleep duration and sleep disturbance were associated with metabolic syndrome, which showed importance of sleep quality and other components for comprehensive understanding of sleep.

The mechanism of the relationship between sleep disorder and metabolic syndrome has not been clarified due to lack of confirming evidence. Sleep rhythm is regulated by hypothalamus [21]. Hypothalamic-pituitary- adrenal axis (HPA) is involved with metabolic syndrome [22], and activated HPA affects sleep disorder [23]. Poor sleep quality and prolonged sleep latency activate HPA, enhancing stress hormone secretion such as cortisol and catecholamine [24,25]. These excess secretions finally leads to increased risk of metabolic syndrome. Sympathomimetic state induced by poor sleep quality reduces leptin level and elevates ghrelin level, which is also related with obesity [4,26]. Additionally, Nguyen-Rodriguez ST et al. have reported that sleep latency has been associated with emotional eating [27]. From the mechanisms mentioned above, it is likely that poor sleep quality, prolonged sleep latency and sleep disturbance increase a risk of metabolic syndrome through sympathomimetic state and emotional eating.

In the present study, short sleep duration was associated with metabolic syndrome in males, but not in females. Some previous studies reported the association between sleep duration and metabolic syndrome [10,28,29]. Our results showed sleep duration was correlated with other PSQI components. Therefore, influence of sleep duration on metabolic syndrome, which was observed in previous studies, was considered smaller than that of global sleep quality.

In this study, habitual sleep efficiency, use of sleep medication and daytime dysfunction showed significant odds ratios, although lacking in reliability. As the participants of the present study were recruited from general population, there were a few individuals who had apparent sleep disorder, including poor habitual efficiency, use of sleep medication and daytime dysfunction. Thus, we need to increase the number of the participants to clarify the relationship between them.

Most previous studies reported that short sleep duration was associated with obesity, hypertension, impaired glucose tolerance and dyslipidemia [10,28,29]. Our study has some advantages. We were able to consider the comprehensive relationships taking lifestyles and depression into account. On the other hand, we were able to evaluate not only the sleep duration but also the comprehensive sleep status including sleep quality, sleep latency, habitual sleep efficiency and sleep disturbance.

However, our study has some limitations. First, this study was limited by the cross-sectional design. Thus, the causal relationship between metabolic syndrome and sleep status could not be revealed. Second, we evaluated sleep-related factors and lifestyles by self-reported brief questionnaires. Sleep evaluation by combining polysomnography and self-report questionnaires may have led to a different result. Additionally, we could have evaluated them with brief questionnaires because complex questionnaires might make the participants confused. Although PSQI focused on short sleep induced by sleep disturbance, short sleepers were a minority in this study. This might tend to give the weaker association between sleep duration and metabolic syndrome compared with previous studies. Third, this study did not exclude obstructive sleep apnea. Obstructive sleep apnea triggers impaired sleep quality and sleep fragmentation. Furthermore, it enhances stress hormone secretion. Fourth, the participants of this study took part in a screening project which was conducted by us. So, we did not deny that there is sampling bias among the participants. Fifth, metabolic syndrome is associated with eating habit, but we did not investigate this because we did not interview the subjects or have suitable questionnaires about eating habit.

## Conclusions

Our results indicated that high global PSQI score was related with high odds ratio of metabolic syndrome in general population. Even when global PSQI score is 5 and less, there is a high odds ratio of metabolic syndrome if they scored high global PSQI score. The PSQI components including poor sleep quality, prolonged sleep latency, short sleep duration and sleep disturbance were also associated with metabolic syndrome.

## Abbreviations

PSQI: Pittsburgh sleep quality index.

## Competing interests

This was not an industry-supported study. This study was supported by a Grant-in-Aid for Young Scientists from The Ministry of Education, Culture, Sports, Science and Technology (MEXT) (21792291) and a Health Science Research Grant from the ministry of Health, Labour and Welfare of the Japanese Government (N20120199). All authors declare that they have no competing interests.

## Authors’ contributions

NO drafted the manuscript and performed the descriptive data analysis. MM participated in the study design and coordination and helped draft the manuscript. IT participated in study design, helped draft the manuscript and reviewed the manuscript for important intellectual content. KS participated in study design, helped draft the manuscript and reviewed the manuscript for important intellectual content. SS participated in the study, helped draft the manuscript and reviewed the manuscript for important intellectual content. NA participated in the study, helped draft the manuscript and reviewed the manuscript for important intellectual content. TU participated in the study design, helped draft the manuscript and reviewed the manuscript for important intellectual content. SN conceived of the study, participated in its design, analysis and interpretation of data and reviewed the manuscript for important intellectual content. All authors read and approved the final manuscript.

## Pre-publication history

The pre-publication history for this paper can be accessed here:

http://www.biomedcentral.com/1471-2458/14/562/prepub
